# Use of Biochar as Filler for Biocomposite Blown Films: Structure-Processing-Properties Relationships

**DOI:** 10.3390/polym13223953

**Published:** 2021-11-16

**Authors:** Luigi Botta, Rosalia Teresi, Vincenzo Titone, Giusi Salvaggio, Francesco Paolo La Mantia, Francesco Lopresti

**Affiliations:** Department of Engineering, University of Palermo, RU INSTM, Viale delle Scienze, 90128 Palermo, Italy; rosalia.teresi@unipa.it (R.T.); vincenzo.titone@unipa.it (V.T.); giusi.salvaggio@community.unipa.it (G.S.); francescopaolo.lamantia@unipa.it (F.P.L.M.); francesco.lopresti01@unipa.it (F.L.)

**Keywords:** biopolymeric blown film, biocomposite film, biochar, photo-oxidative resistance

## Abstract

In this work, biocomposite blown films based on poly(butylene adipate-co-terephthalate) (PBAT) as biopolymeric matrix and biochar (BC) as filler were successfully fabricated. The materials were subjected to a film-blowing process after being compounded in a twin-screw extruder. The preliminary investigations conducted on melt-mixed PBAT/BC composites allowed PBAT/BC 5% and PBAT/BC 10% to be identified as the most appropriate formulations to be processed via film blowing. The blown films exhibited mechanical performances adequate for possible application as film for packaging, agricultural, and compost bags. The addition of BC led to an improvement of the elastic modulus, still maintaining high values of deformation. Water contact angle measurements revealed an increase in the hydrophobic behavior of the biocomposite films compared to PBAT. Additionally, accelerated degradative tests monitored by tensile tests and spectroscopic analysis revealed that the filler induced a photo-oxidative resistance on PBAT by delaying the degradation phenomena.

## 1. Introduction

In recent years, polymers and polymer-based systems have been widely investigated as materials for thin-film preparation [1,2]. Usually, thermoplastic polymers offer low raw material costs and a well-established manufacturing process that can be easily scaled to large-scale production [3]. However, major concerns for traditional thermoplastics used for film preparation, commonly derived from fossil fuels, refer to the non-renewability and non-biodegradability of the raw materials chosen for their production, thus causing environmental issues [4,5]. Due to the rising attention towards eco-sustainable products, it is unsurprising that more and more research groups and industries are exploring the possibility of using new biodegradable and compostable polymers suitable for different applications [6,7,8,9,10,11].

A wide plethora of biodegradable polymers, such as polysaccharides, proteins, and lipids, were proposed as suitable materials for thin-film preparation [6,12,13]. In this context, poly(butylene adipate-co-terephthalate) (PBAT) is an aliphatic/aromatic copolyester that is biodegradable and compostable and, due to its interesting properties, is considered among the most promising biopolymers for packaging film, agricultural film, and compost bag fabrication [14,15,16,17].

Film blowing is considered the most popular approach for fabricating polymer-based films. However, this technique can be difficult to apply for biopolymers due to their typical reduced elongation and low melt strength compared to non-biodegradable polymers [18,19,20]. These characteristics can affect the bubble making it wrinkled and unstable during the film-blowing operations [21]. Moreover, bioplastics often highlight drawbacks such as poor mechanical properties, high wettability, and low durability [22,23,24].

In order to overcome these issues, several approaches can be considered, such as improving the material melt strength by adopting viscosity enhancers [25], or the addition of different kinds of fillers likely able to concurrently improve the final properties as well as the processability of the blown films [14,15,26,27,28]. Both of these methods permit the mechanical and physical properties of biopolymers to be controlled in a relatively simple and potentially industrially scalable way [29,30]. However, depending on the kind of chosen filler, this approach could question the environmental sustainability of the polymer matrix as well as its processability. Therefore, inexpensive, non-toxic, and eco-friendly fillers derived from biomass can be considered among the most promising additives for eco-sustainable biopolymer green biocomposites [29,30].

In this context, biochar (BC) is attracting high interest as filler for polymer-based composites [30,31,32,33,34] due to its challenging properties, such as high thermal and chemical stability combined with its cost-effectiveness and eco-sustainability [35,36]. BC is usually produced by the pyrolysis of wastes from the forestry and agricultural industries [32], and its structure can be modified by tuning the pyrolysis conditions [35].

Recently, several remarkable articles about the efficacy of BC as filler for the fabrication of green composites were published [37,38,39,40,41]. However, to the best of our knowledge, no paper has ever dealt with the effect of biochar on the processability and properties of PBAT-based blown films.

Therefore, in this work, biocomposites based on PBAT and biochar were first compounded in a twin-screw extruder and then subjected to a film-blowing process. As a preliminary investigation, systematic morphological, thermal, mechanical, and rheological characterizations of the materials prepared by melt mixing and compression molding were performed, aiming at obtaining a detailed frame of the biocomposite processing behavior. In particular, the effect of the filler amount on the melt rheological behavior in non-isothermal elongational flow was investigated in order to assess the adequate filler concentrations for film-blowing processing.

The produced biocomposite blown films were characterized by tensile tests, contact angle, and spectrophotometric measurements. Finally, since BC is supposed to somehow affect the photo-oxidative behavior of PBAT, the films were subjected to artificial exposure to UV-B light. The photo-oxidation was followed by monitoring the change in the mechanical and spectroscopic properties of the blown films at different irradiation times.

## 2. Materials and Methods

### 2.1. Materials

PBAT (ecoflex^®^ F Blend C1200, Basf, SE, Ludwigshafen, Germany) is a film grade with a melt flow rate (MFR) of 2.7–4.9 g/10 min (190 °C, 2.16 kg), a density in the range of 1.25–1.27 g/cm^3^, and a melting temperature in the range of 110–120 °C.

Commercial biochar powder (hereafter coded as BC) used in the food industry (Spigadoro, Perugia, Italy) was chosen as filler. In particular, as reported in the technical data sheet of the supplier, this biochar was obtained from the pyrolysis of birch and beech wood.

In order to avoid hydrolytic scission of PBAT during processing, the PBAT and biochar were dried under vacuum overnight at 70 °C and at 105 °C, respectively.

### 2.2. Processing

For the preliminary investigations, PBAT/BC composites were prepared by melt mixing using a Brabender batch mixer (Brabender, Model PLE330, Duisburg, Germany).

In brief, PBAT and 5 wt%, 10 wt%, or 20 wt% of BC particles were pre-mixed in solid state and then fed to the batch mixer. The process was performed according to the following operative parameters: T = 170 °C, rotor speed n = 60 rpm for a time t = 5 min. During the process, the torque was measured as a function of time and then the blend was rapidly cooled in liquid nitrogen.

Samples of different thicknesses were prepared by compression-molding using a laboratory press (Carver, Wabash, IN, USA) at 170 °C for 120 s at a pressure of 100 bar. Pure PBAT was subjected to the same processes and used as the control sample.

Blown films with the same filler concentration were obtained by double extrusion. First, the materials were extruded through a rotating twin-screw extruder (OMC, Saronno, Italy) with the following temperature profile: 150–160–160–170–180 °C and screw speed of 180 rpm.

Film blowing of the extruded PBAT/BC pellets was carried out in a single screw extruder (D = 19 mm, L/D = 25) Brabender (type 832004, Duisburg, Germany) at a temperature profile of 150–160–160 °C, die temperature of 180 °C, and screw speed set at 80 rpm.

### 2.3. Characterization Techniques

Rheological characterization in shear flow was performed by using a Rheologic 1000 (Ceast, Torino, Italy) capillary rheometer equipped with the following capillary geometry: a diameter equal to 1 mm and a length on bore diameter ratio equal to 40.

The same capillary rheometer with a tensile drawing unit tool was used to determine the rheological behavior of the biocomposites in non-isothermal elongational flow. More in detail, the force at break applied to the molten filament, i.e., the melt strength (MS), as well as the drawing speed at breaking and the extrusion rate ratio, i.e., the breaking–stretching ratio (BSR), were evaluated. The temperatures selected for the rheological characterization corresponded to the temperature achieved in the die during the film blowing process, i.e., 170 °C.

The morphology of all the materials was observed using a Quanta 200 ESEM, FEI (Hillsboro, OR, USA) scanning electron microscope (SEM). For the PBAT-based biocomposites, each specimen was fractured in liquid nitrogen and the fracture surface was analyzed.

Differential scanning calorimetry (Setaram, model DSC131, Lyons, France) was carried out to assess the thermal characterization of the materials. The analyses were performed in the presence of inert gas (nitrogen). Samples of similar weight (~5 mg) were subjected to heating/cooling/heating ramps with a scanning speed of 10 °C/min in the 30–190 °C temperature range.

The degree of crystallinity (χ) of PBAT was calculated according to Equation (1) [23]:

(1) $$\chi \;\left(\%\right)=\frac{\Delta H_{m}}{\Delta H^{0}{}_{\mathrm{PBAT}}\times X_{\mathrm{PBAT}}}\times 100$$

where ΔH_m_ is the melting enthalpy evaluated by the calorimeter, X_PBAT_ represents the PBAT weight fraction, and ΔH^0^_PBAT_ is the melting enthalpy of 100% crystalline PBAT (114 J/g) [42].

Static water contact angle (WCA) measurements were carried using an FTA 1000 (First Ten Ångstroms, Portsmouth, VA, USA) analyzer. More in detail, a droplet (~5 µL) of deionized water was positioned on the PBAT-based blown-film surface and maintained for 10 s before the image capture. At least seven droplet images from each composite sample were analyzed.

The PBAT-based film’s optical properties were investigated by analyzing the light transmittance through the samples in the UV-vis region (200–800 nm) by using a UV–vis spectrophotometer (model UVPC 2401, Shimadzu Italia s.r.L., Milan, Italy).

A Q-UV chamber (Q-Labs Corp., Westlake, OH, USA) containing eight UVB-313 lamps was used to assess the photo-oxidative resistance of the samples. The weathering conditions were chosen according to a modified ISO 4892-3 method. In particular, the exposure cycle conditions were 8 h of light at 55 °C followed by 4 h of condensation at 45 °C and relative humidity of 40 ± 3%.

In order to follow the chemical changes in PBAT-based blown films during the accelerated weathering, an FTIR/ATR analysis (Perkin-Elmer FT-IR/NIR Spectrum 400, Waltham, MA, USA) was carried out. Four accumulation scans with a resolution of 4 cm^−1^ in the range of 4000–400 cm^−1^ were collected for each sample.

Tensile mechanical measurements were assessed using an Instron 3365 (Instron, Norwood, MA, USA) universal testing machine equipped with a 1 kN load cell. The measurements were performed at 1 mm/min for 1 min; thereafter, the crosshead speed was changed to 100 mm/min until sample fracture. The tests were performed on rectangular film specimens (10 mm × 90 mm) and fixed to Instron gauges 30 mm from each other. The effective thickness of each sample was measured before the test. The elastic modulus (E) was calculated as the slope of the initial part of the engineering stress–strain curves. For each material, at least seven specimens were tested for both non-photo-oxidized and photo-oxidized films at different time points.

## 3. Results and Discussion

### 3.1. Preliminary Investigation

To fabricate the biocomposite blown films, a preliminary investigation was assessed in order to find the optimal BC concentration for PBAT/BC processing and properties. At this stage, achieving adequate viscosity, a good level of particle dispersion, and adequate mechanical properties are some of the key parameters for processing success.

**Figure 1:**
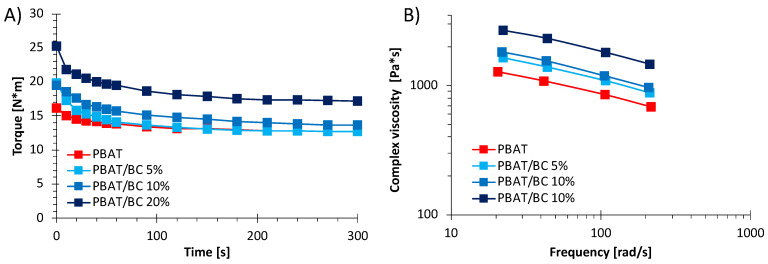
(**A**) Torque as a function of time and (**B**) complex viscosity as a function of the frequency of PBAT and PBAT/BC biocomposites.

Figure 1A shows the torque curves recorded during melt mixing at 170 °C at 60 rpm of both pure PBAT and PBAT/BC biocomposites. The torque behavior during mixing provided an indirect measure of the melt viscosity and, as a consequence, information about the polymeric system processability [43].

The torque curves clearly revealed that after about one minute, a plateau was reached for all the systems analyzed, thus indicating the effective and complete mixing of the system [44]. The presence of the filler affected the torque values that increased upon increasing the concentration of BC added. This result was expected since it is well known that solid particles tend to increase the viscosity of polymer melts [9]. In fact, the addition of 20 wt% of BC caused a steep increase in the torque values, which were higher than those of PBAT during the processing time. Differently, the PBAT/BC 5% torque was quite similar to that of pure PBAT and perfectly stackable to PBAT after the first minute of processing.

Figure 1B shows the flow curves of the PBAT and PBAT/BC systems, highlighting that the rheological responses of PBAT-based biocomposites were significantly affected by the presence of the filler. More in detail, as the concentration of the filler increased, the viscosity of the melt increased compared to the pure matrix, without significantly altering the trend of the viscosity as a function of frequency. Interestingly, the PBAT/BC 5% and PBAT/BC 10% systems showed flow curves comparable to each other, whereas the addition of 20% BC caused a much greater increase in viscosity compared to the other two biocomposites. The achievement of higher shear viscosity values with respect to PBAT in the whole range of frequencies investigated suggests an influence of the embedded filler on the long-range and short-range dynamics of polymer chains [3].

Information about the biocomposite rheological behavior when subjected to non-isothermal elongational flow was of primary importance to assess the processability in film blowing of the formulated systems [45].

**Figure 2:**
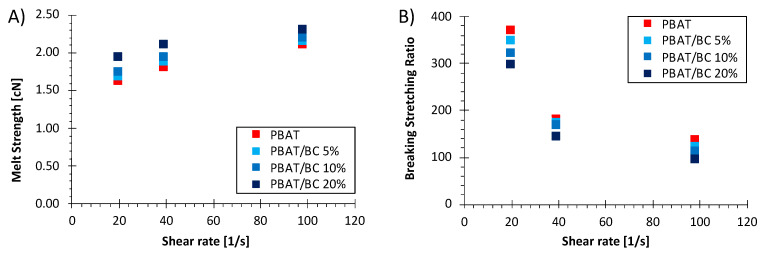
(**A**) Melt strength and (**B**) breaking–stretching ratio as a function of the apparent shear rate of PBAT and PBAT/BC biocomposites.

For this reason, for all investigated systems, the melt strength (MS) and the breaking stretching ratio (BSR) were evaluated as a function of the applied shear rate, as shown in Figure 2A,B, respectively. In most cases, a higher viscosity of the melt systems leads to higher values of MS and, as a consequence, higher workability [45]. As expected, the MS increased upon increasing the filler concentration in the polymer matrix. This result can be ascribed to an increased resistance to the elongational flow as a consequence of the solid-phase addition. Coherently to the viscosity results, the greatest increase in MS occurred by adding 20 wt% biochar to the polymer matrix. On the other hand, the BSR values decreased upon increasing the BC concentration for all shear rates investigated. In particular, the biocomposites incorporating the highest BC content exhibited the lowest values of BSR. This result suggests that the PBAT/BC 20% formulation was inadequate for being processed via film blowing.

**Figure 3:**
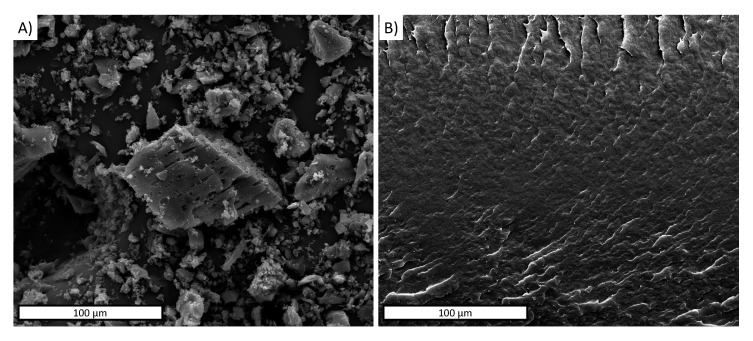
SEM micrographs of (**A**) biochar particles and (**B**) PBAT nitrogen-fractured surface.

SEM analysis was carried out in order to investigate the morphology of both the BC microparticles and PBAT surface (Figure 3A,B). An SEM micrograph of BC, reported in Figure 3A, displayed porous and nearly parallelepipedal particles in a variable granulometry in the range of 5–65 μm. Figure 3B reports the SEM image of the PBAT nitrogen-fractured surface, characterized by a smooth and homogeneous morphology.

**Figure 4:**
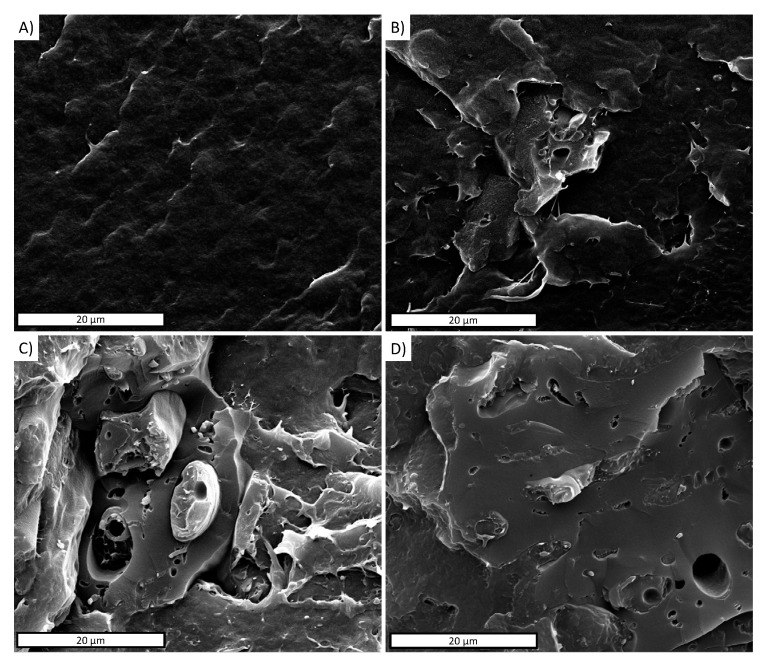
Nitrogen-fractured surface SEM micrographs of (**A**) PBAT; (**B**) PBAT/BC 5%; (**C**) PBAT/BC 10%; (**D**) PBAT/BC 20%.

Figure 4A–D shows the micrographs of the nitrogen-fractured surface of the PBAT and PBAT/BC composites prepared in this work.

The PBAT cross-section in Figure 4A appears smooth and homogeneous. Regardless of the BC concentration, the biocomposites exhibit a uniform dispersion of the embedded filler, although the particle size inhomogeneity is clearly visible (Figure 4B–D).

**Figure 5:**
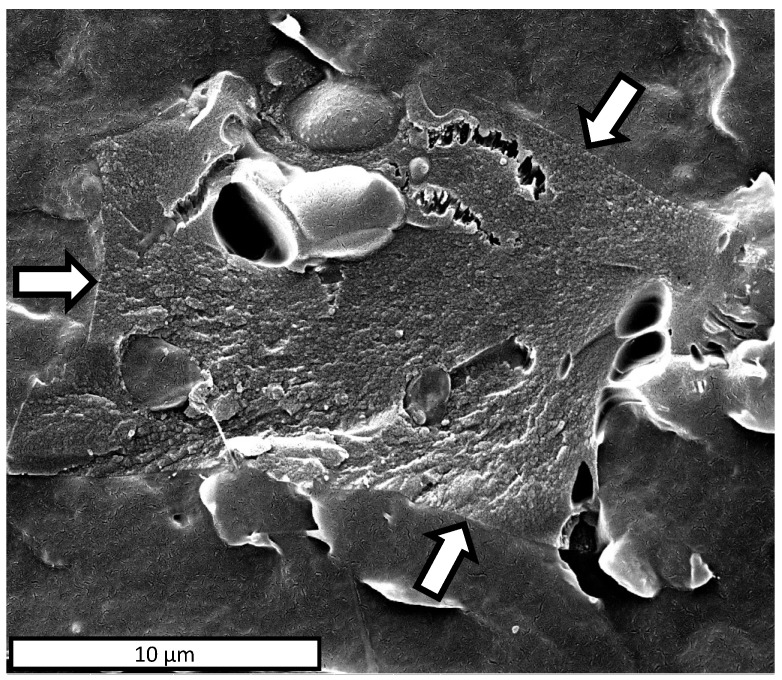
SEM micrographs of the PBAT–BC interface in a PBAT/BC 5% biocomposite. The arrows indicate the PBAT-BC interface.

The morphology of the fractured surfaces of the PBTA/BC composites suggests good matrix/filler interfacial adhesion. In particular, the close-up on the PBTA/BC interface, highlighted by the arrows in Figure 5, permits the good adhesion between the filler and matrix to be better observed. This result can likely be ascribed to the affinity existing between the matrix and biochar and it was found to be independent of the filler concentration (data not shown for the sake of brevity). The high magnification of Figure 5 permits the BC porous structure to be better appreciated. The elongated and rough pores can be ascribed to the retainment of the original skeleton of wood [46]. The smooth and oval pores were already observed in previous works and related to the rapid release of gaseous products during the pyrolysis [46].

**Figure 6:**
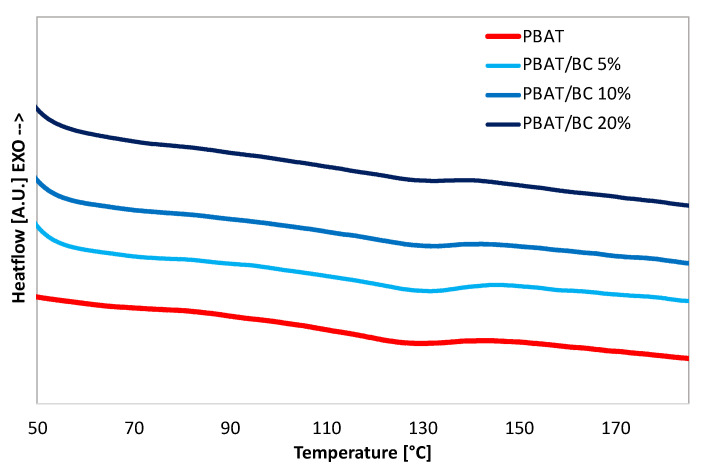
Differential scanning calorimetry (DSC) thermograms recorded during the second heating scan.

Figure 6 shows the thermograms recorded by DSC during the second heating scan of the compression-molded samples. PBAT showed the typical thermogram of an almost amorphous polymer characterized by a small endothermic peak at 129.5 °C due to the polymer melting phenomenon. Coherently, the PBAT crystallinity, reported in Table 1, was 7%. Regardless of the BC concentration, Table 1 highlights that the addition of the filler involved a minimum decrease in the percentage crystallinity compared to pure PBAT, although a slight increase in Tm from 129.5 °C of PBAT to 133 °C of PBAT/BC 20% can be observed.

Therefore, the DSC results revealed that BC did not substantially influence PBAT structure, which remained almost amorphous despite the filler addition. This result was expected since PBAT is a random copolymer characterized by low crystallinity due to its intrinsic irregularity in structure, which inhibits high crystallinity [47]. However, the slight increase in Tm confirmed that BC and PBAT were compatible to some extent, as reported in previous researches [48].

**Figure 7:**
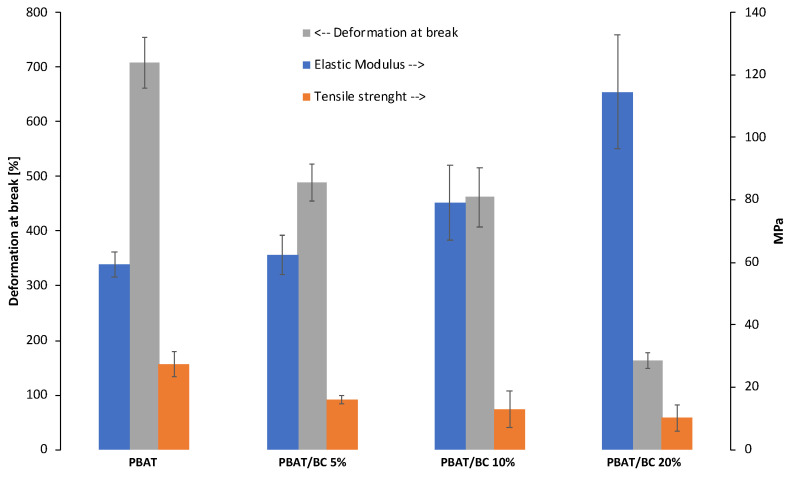
Histogram of the elastic modulus, tensile strength, and deformation at break of PBAT-based casted samples.

Figure 7 summarizes the tensile properties of neat PBAT and PBAT/BC samples obtained by compression molding. The histogram highlights that the addition of 5 wt% of filler did not affect the elastic modulus of the material, whereas the moduli of PBAT/BC 10% and PBAT/BC 20% biocomposites increased by about 33% and 93%, respectively, with respect to the pure biopolymer. The elastic modulus increase of the composites was probably due to the reinforcing effect of BC on the matrix due to the good adhesion observed by SEM analysis, since the crystallinity of the samples was not affected by the filler.

On the other hand, the presence of BC led to a reduction in the tensile strength of all the composites compared to PBAT (TS_PBAT_ = 27.3 MPa). More in detail, PBAT/BC 5% showed a TS value around 16 MPa, about 40% lower than that of PBAT. At a higher filler concentration, the TS linearly decreased to about 10 MPa for PBAT/BC 20% biocomposite, i.e., about 60% lower than PBAT.

Similarly, the addition of BC caused a reduction in the elongation at break of all the biocomposites investigated in this work. More precisely, the elongation at break of PBAT was 710%, whereas PBAT/BC 5% and 10% exhibited a reduction of about 31% and 35% of this value, respectively. The lowest elongation, around 160%, was recorded for the PBAT/BC 20% biocomposite.

The decrease in the tensile strength of the biocomposites can be ascribed to the premature failure of the samples, which is expected when rigid particles are loaded in polymer matrices. Moreover, it is well known that despite the good matrix–filler adhesion observed via SEM analysis (Figure 5), the filler–matrix interface as well as the filler–voids can act as stress concentrators in composites [49].

### 3.2. Characterization of PBAT/BC Blown Films

This preliminary study was carried out in order to identify the adequate compositions for processing by film blowing.

SEM analysis revealed that regardless of the BC percentage added to the polymer matrix, all formulated composites were characterized by good interfacial adhesion. However, the rheological measurements revealed that an addition of 20 wt% of BC into the polymer matrix led to an excessive increase in the melt viscosity that compromised the film-blowing processing. Furthermore, the deformation at break of the PBAT/BC 20% systems was drastically lower than that of the other composites.

Therefore, the film-blowing processing was successfully carried out only on the PBAT, PBAT/BC 5%, and PBAT/BC 10% formulations.

#### 3.2.1. Contact Angle Measurements

Scientific literature reports that low wettability of films reduces their capacity to adsorb water from environmental moisture, which is a key parameter for food packaging applications [50,51].

**Figure 8:**
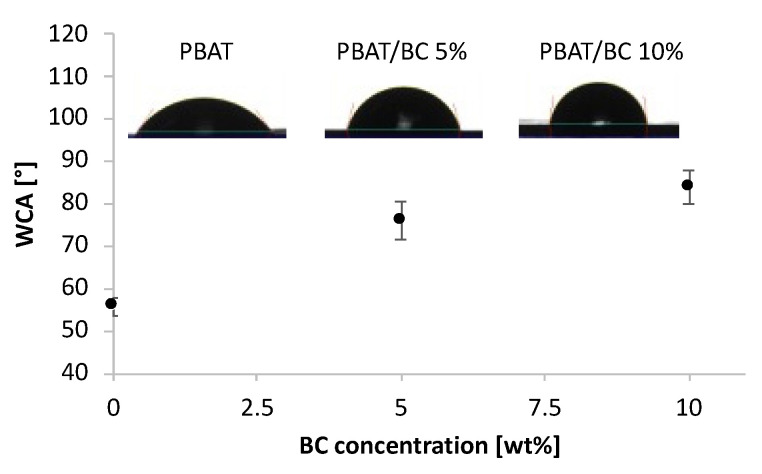
Water contact angles of PBAT, PBAT/BC 5%, and PBAT/BC 10%.

The surface wettability of the films was analyzed to evaluate the hydrophilic/hydrophobic character of the PBAT-based films through static water contact angle measurements (WCA) (Figure 8).

PBAT films showed intrinsic hydrophilicity displaying a WCA value around 55°, whereas the addition of BC induced a noticeable increase in this value. More in detail, the WCA of PBAT/BC 5% and PBAT/BC 10% films was found to be equal to 76° and 84°, respectively.

In order to explain these results, it can be considered that the wettability of polymeric films is strongly dependent not only on the surface topographical properties but also on the chemical properties of the filler [52]. BC is obtained by thermochemical conversion processes, which involves the loss of the hydrophilic groups of the lignocellulosic structures of the plant biomass of origin, thus explaining the WCA results [31].

#### 3.2.2. UV-Vis Characterization

**Figure 9:**
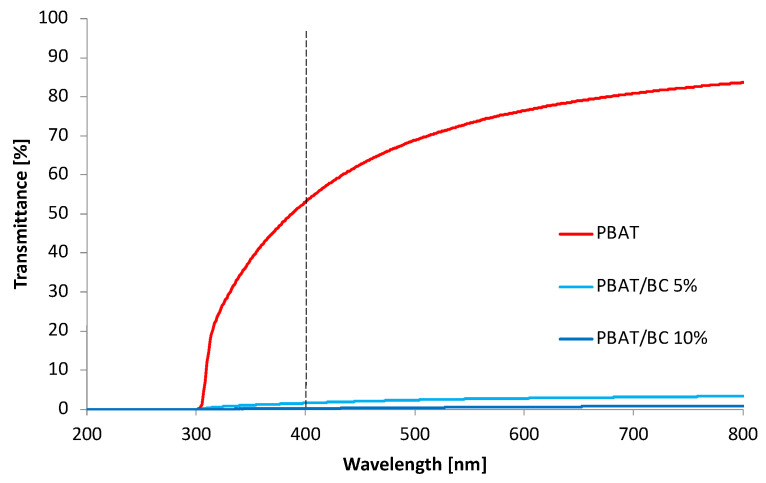
UV-Vis spectra of PBAT-based blown film.

PBAT is characterized by high transparency due to its very low crystallinity degree, as confirmed by the high transmittance recorded by UV-Vis measurements in the range of 400–800 nm (Figure 9). On the other hand, PBAT showed poor transmittance to UV radiation in the range of 300–400 nm. Regardless of the concentration of BC added to the polymer matrix, the film transparency strongly decreased, leading the transmittance in the visible range close to zero. Interestingly, the transmittance in the UV range also drastically decreased when BC was added to the polymer matrix.

#### 3.2.3. PBAT/BC Blown-Film Mechanical Properties

**Figure 10:**
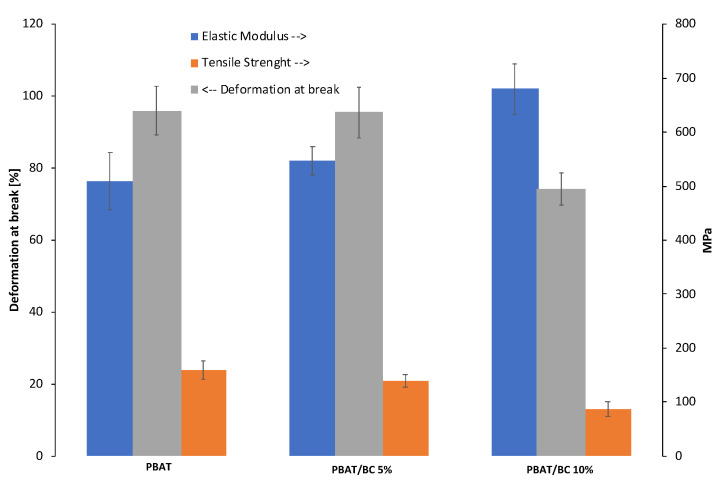
Histogram of the elastic modulus, tensile strength, and deformation at break of PBAT-based blown film.

Mechanical characterization of PBAT and PBAT/BC blown film was conducted on samples in the machine direction (Figure 10).

The tensile properties of PBAT films highlighted the achievement of a higher elastic modulus compared to the isotropic casted samples (whose tensile properties are shown in Figure 7). This finding, as confirmed by the lower ductility, may be related to the effect of the elongational flow on the materials during the processing, which could have induced a preferential orientation of the polymeric chains along with the flow direction [3]. As expected, and coherent to the mechanical results obtained by casted samples, the elastic modulus of the blown film increased upon increasing the filler content. In this case, the elastic modulus increment was around 7% and 33% higher than that of PBAT films for PBAT/BC 5% and PBAT/BC 10%, respectively. Unlike the casted samples, the tensile strength and the elongation at break of the PBA/BC 5% biocomposites remained comparable to those of the pure blown matrix. Furthermore, in the case of PBAT/BC 10%, decrease in the tensile strength and elongation at break was lower than that observed for the casted sample with the same BC concentration. This result can likely be ascribed to the manufacturing process itself and to the orientation of the filler in the matrix during blow molding. Nevertheless, the biocomposite blown film maintained enough elongation at break for packaging, agricultural, and compost bag applications.

#### 3.2.4. Effect of Photo-Oxidation on the Mechanical Properties of PBAT/BC Blown Films

**Figure 11:**
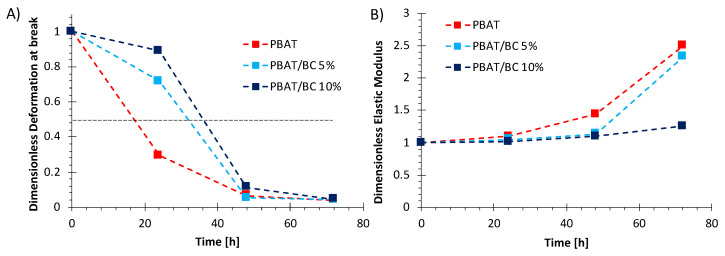
Dimensionless values of (**A**) deformation at break and (**B**) elastic modulus of PBAT-based blown film as a function of photo-oxidation time.

Adequate photo-oxidative stability is a key parameter for a film designed for agriculture applications [53]. Therefore, the mechanical properties of the PBAT-based film as a function of the exposure time to photo-oxidation were evaluated and are reported in Figure 11A,B. More in detail, Figure 11A,B reports the dimensionless deformation at break and dimensionless elastic modulus, respectively, obtained as the ratio between the values at a given irradiation time point and the value of the corresponding samples before exposure to photo-oxidation. In particular, elongation at break is the main mechanical parameter affected by the polymers’ structural and morphological changes after exposure to photo-oxidation [54]. The elongation at break–exposure time curves (Figure 11A) displayed a significant decrease of deformation at break for PBAT already after 24 h of photo-oxidation, thus highlighting a neat mechanical behavior change in the polymer matrix from ductile to brittle.

On the other hand, PBAT/BC 5% and PBAT/BC 10% films showed higher deformation at break after exposure to photo-oxidation that can be better monitored by observing the half time of the elongation at break. The half time of the elongation at break is a parameter evaluated as the time point at which the elongation at break value is half the initial one [54]. This parameter is considered the maximum time at which the film can be still used [54]. As can be seen in Figure 11A, the half time of PBAT was 17 h, whereas it was found to be equal to 32 h and 36 h for PBAT/BC 5% and PBAT/BC 10%, respectively. Therefore, BC succeeded in increasing the photo-oxidation resistance of PBAT and to more than doubling the half time of the films in the case of PBAT/BC 10%.

On the other hand, the elastic modulus (Figure 11B) increases upon increasing the exposure time for all the samples, as has been observed in other semicrystalline polymers [55]. However, in the case of PBAT, the increase can be attributed to the formation of crosslinks of the amorphous phase that make the polymer matrix more rigid, as observed in a previous work [55]. Interestingly, the elastic modulus increase as a function of photo-oxidation time for PBAT/BC 5% and PBAT/BC 10% was clearly lower than in the case of pure PBAT films, in particular after 72 h for the composite blown film containing 10 wt% BC.

**Figure 12:**
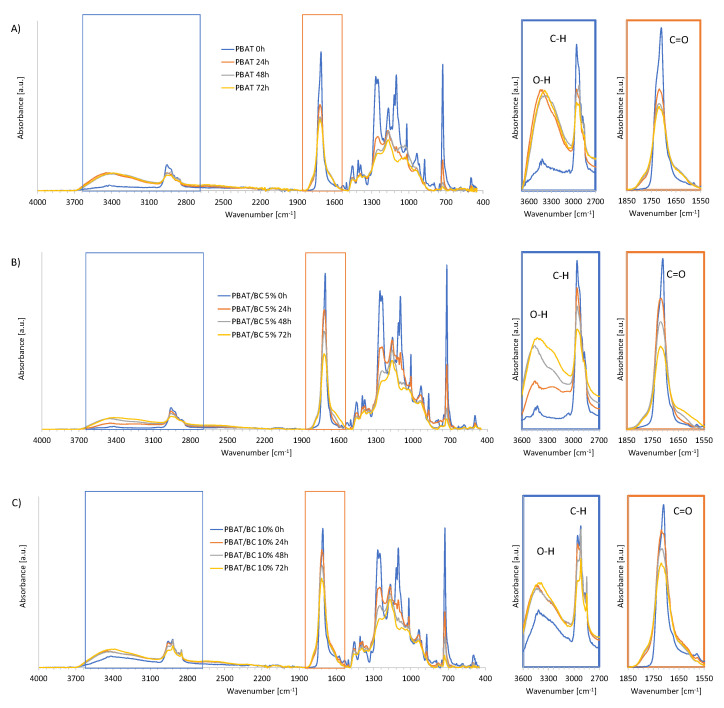
FTIR spectra as a function of photo-oxidation time of (**A**) PBAT, (**B**) PBAT/BC 5%, and (**C**) PBAT/BC 10% blown films.

Another effective approach to monitoring photochemical transformations occurring on polymer matrices during UV irradiation is FTIR-ATR analysis. The FTIR-ATR spectra collected for the PBAT-based blown films at the different photo-oxidation time points are shown in Figure 12A–C. The insets in the same figures represent a zoom of two spectral regions of interest for highlighting the modifications that occurred on the main bands related to the degradation of the polymer matrix.

In all the spectra, the typical bands of PBAT can be easily recognized. More in detail, -OH vibrations at ca. 3400–3450 cm^−1^ are clearly visible, whereas bands at 2957 cm^−1^ and 2874 cm^−1^ should be assigned to a CH_2_ stretching mode. The carbonyl groups (C=O) presented a strong peak around 1710 cm^−1^, with a sharp peak representing CH_2_-groups at 720 cm^−1^. The C–O bond in the ester linkage was observed at around 1275–1250 cm^−1^ [56,57]. However, all these signals proved to change their intensity as a function of exposure time, indicating evidence of photodegradation and photo-oxidation reactions. More in detail, in Figure 11A PBAT blown films showed a reduction and a broadening of the carbonyl peak (C=O). The left shoulder (1790–1750 cm^−1^) indicates the formation of free C=O, and the right shoulder (1590–1630 cm^−1^) represents the formation of a lower-molecular-weight ester. These observations indicate a Norrish I chain scission reaction [58].

Furthermore, in Figure 12A the formation of hydrogen-bonded OH at 3530 cm^−1^, free OOH (or peroxide) at 3440 cm^−1^, and the broad peak at 3370 cm^−1^ of the polymer OH all indicate a photo-oxidative reaction, and were formed by an autocatalytic reaction of the carboxylic terminal groups from the Norrish II reaction [58]. These chemical modifications led to chain scissions that can be addressed for the reduction of elongations at break. Interestingly, the FTIR-ATR spectra modifications induced by the UV exposure in the case of PBAT/BC 5% (Figure 12B) and PBAT/BC 10% blown films (Figure 12C) were found to be less intense than for PBAT blown films, in particular after 24 h. These results corroborated the hypothesis that BC induces a photo-oxidative resistance on PBAT by delaying the degradation phenomena of the polymer matrix.

## 4. Conclusions

In this work, the properties and the filmability of biocomposite materials based on PBAT as polymer matrix and biochar as filler were investigated.

All the PBAT/BC composites showed uniform filler dispersion and good adhesion within the selected biopolymeric matrix, which resulted in an increase in the elastic modulus.

However, the PBAT/BC 20% samples showed a dramatic reduction in the elongation at break and a decrease in the BSR.

These preliminary results led to PBAT/BC 5% and PBAT/BS 10% being selected as the most suitable composites to be processed via film blowing.

Therefore, PBAT/BC 5% and 10% were successfully produced through film blowing and they exhibited appropriate performances for their possible use as materials for packaging, agricultural, and compost bag applications. In fact, the elastic modulus increased upon increasing the BC concentration, still maintaining high values of deformation at break and an improved hydrophobic behavior compared to PBAT. Additionally, the presence of the filler induced a photo-oxidative resistance on PBAT by delaying the degradation phenomena of the polymer matrix. This result was even more notable for PBAT/BC 10% than for PBAT/BC 5% and enables potential open-air applications of the PBAT-based films.

## Figures and Tables

**Table 1:** Thermal properties collected during the second heating scan for all investigated systems.

Sample Name	ΔH (J/g)	χ (%)	T_m_ (°C)
PBAT	8.04	7	129.5
PBAT/BC 5%	5.97	5.2	131.2
PBAT/BC 10%	5.90	5.1	132.7
PBAT/BC 20%	7.11	6.2	133.0

## Data Availability

The data presented in this study are available on request from the corresponding author.
